# One Giant Leap from Mouse to Man: The Microbiota–Gut–Brain Axis in Mood Disorders and Translational Challenges Moving towards Human Clinical Trials

**DOI:** 10.3390/nu14030568

**Published:** 2022-01-27

**Authors:** Sofia D. Forssten, Arthur C. Ouwehand, Síle M. Griffin, Elaine Patterson

**Affiliations:** IFF Health and Biosciences, Danisco Sweeteners Oy, Sokeritehtaantie 20, 02460 Kantvik, Finland; Arthur.Ouwehand@iff.com (A.C.O.); silemgriffin@gmail.com (S.M.G.); elainepatterson01@gmail.com (E.P.)

**Keywords:** probiotic, gut microbiota, neurotransmitters, anxiety, depression

## Abstract

The microbiota–gut–brain axis is a bidirectional communication pathway that enables the gut microbiota to communicate with the brain through direct and indirect signaling pathways to influence brain physiology, function, and even behavior. Research has shown that probiotics can improve several aspects of health by changing the environment within the gut, and several lines of evidence now indicate a beneficial effect of probiotics on mental and brain health. Such evidence has prompted the arrival of a new term to the world of biotics research: psychobiotics, defined as any exogenous influence whose effect on mental health is bacterially mediated. Several taxonomic changes in the gut microbiota have been reported in neurodevelopmental disorders, mood disorders such as anxiety and depression, and neurodegenerative disorders such as Alzheimer’s disease. While clinical evidence supporting the role of the gut microbiota in mental and brain health, and indeed demonstrating the beneficial effects of probiotics is rapidly accumulating, most of the evidence to date has emerged from preclinical studies employing different animal models. The purpose of this review is to focus on the role of probiotics and the microbiota–gut–brain axis in relation to mood disorders and to review the current translational challenges from preclinical to clinical research.

## 1. Introduction

The gut microbiota constitute a so-called virtual organ consisting of a complex ecosystem involving around one hundred trillion microorganisms, mostly consisting of bacteria, but also including viruses, fungi, and protozoa [1,2]. In humans, the caecum and distal colon are the sites of highest microbial biomass, with about 95% of gut microbes located there, while the small intestine makes a numerically lesser, although functionally considerable, contribution [3]. The host and the gut microbiota have complex interactions that are affected through different aspects of metabolism. The gut microbiota break down complex carbohydrates and proteins, while producing metabolites that have either a positive or negative impact on the host [4,5,6]. Microbial communities within the gut change in composition, diversity, and activity across the lifespan, which also has a lifelong impact on neurophysiology and behavior through the multifaceted relationship with the host [7,8]. 

The gut–brain axis consists of a bidirectional communication pathway between the central nervous system (CNS) and the enteric nervous system (ENS), linking the cognitive and emotional centers of the brain with peripheral intestinal functions. Thus, the microbiota of the intestinal lumen affect the CNS activities of the host, such as cognition and the stress response, and likewise the activity of the brain affects microbial composition. Recent advances in research have described the importance of the gut microbiota in influencing these interactions; thus, the microbiota–gut–brain axis is a more relevant term to describe this bi-directional communication pathway [9]. 

The balance between the human microbiome and the development of psychopathologies is interesting, since the gut microbiota can be altered through external factors such as diet, probiotics, prebiotics, and antibiotics, all of which have been demonstrated to affect brain functions and behavior. Probiotics are defined as “live microorganisms that, when administered in adequate amounts, confer a health benefit on the host” [10]. More recently, the term psychobiotic was coined to describe any exogenous influence (e.g., probiotics) whose positive effect on mental health is bacterially mediated [11,12]. Current probiotics belong mainly to the genera *Lactobacillus* (*sensu lato*) and *Bifidobacterium*, although strains from other genera, such as *Saccharomyces* and *Bacillus*, are also commercialized. Single and multi-strain probiotic intervention studies have demonstrated beneficial effects [13] for several conditions, such as constipation [14], allergy [15], antibiotic associated diarrhea [16], and modulation of the immune system [17]. In addition, accumulating evidence now supports the role of probiotics in mental and brain health [18], as described below. This review will focus on the role of probiotics in modulating the microbiota–gut–brain axis to impact mood and behavior, as well as the translational challenges moving from preclinical to clinical intervention studies, published up to December 2021. 

## 2. Pathways of Communication along the Microbiota–Gut–Brain Axis 

The microbiota–gut–brain axis is a complex network of different communication pathways within the endocrine system, the hypothalamic–pituitary–adrenal (HPA) axis, the ENS, and the immune system. Regarding co-metabolism, the concept of a “leaky gut” may also play a role in the movement of metabolites. The microbiota–gut–brain axis does not solely relate to any single one of these communication pathways, but each plays an essential role. 

### 2.1. The Autonomic Nervous System and the Enteric Nervous System

The autonomic nervous system (ANS) regulates the unconscious control of physiological homeostasis. The ENS is a network of around 500 million neurons at the interface of the gut microbiota and the host, lining the entire intestinal tract from the esophagus to the anus and which is part of the ANS. The ENS responds to receptor input from the intestine and via ganglia within the spinal cord and the brain’s medulla to coordinate various intestinal functions, such as smooth muscle activity, glandular secretion, and sphincter control [19]. Although intestinal functionality is regulated to maintain homeostasis, adaptation is possible for environmental conditions such as stress [19]. The ENS connects to the CNS (including the brain) through the vagus nerve [20], thus allowing the brain to sense the environment within the gut.

### 2.2. The Vagus Nerve

The vagus nerve is the most direct route of communication between the gut and the brain, thus enabling bidirectional communication. The vagus nerve has also been indicated in the etiology of Parkinson’s disease [21], Alzheimer’s disease [22], and depression [23]. What these conditions have in common is that they are influenced by the gut microbiota through their metabolites and immune-modulating activity. The vagus nerve functions both as a signaling pathway and transfers metabolites and other components to the brain [21]. The vagus nerve responds to components produced or induced by the gut microbiota, such as short chain fatty acids (SCFA), endotoxins, peptides, and cytokines [20]. Furthermore, neurotransmitters such as serotonin produced within the gut influence vagal functionality [23].

### 2.3. Immune Signaling

The gut microbiota are essential for the healthy development and function of the peripheral immune system and for the development and maturation of the innate immune cells of the brain (reviewed in [24]). Within the intestine, the mucosa provides the barrier between the inside ‘self’ and outside ‘non-self’, consisting of digesta and resident and in-coming microbes. Inflammatory responses and impairment of intestinal barrier function often go hand in hand. The induction of proinflammatory cytokines, such as interleukin (IL)-1β, IL-6, IL-18, and tumor necrosis factor (TNF)-α, have all been associated with depression [20,25] and increased peripheral inflammation has been observed in many psychiatric diseases such as depression, anxiety, and even autism spectrum disorder (reviewed in [26]). Circulating cytokines can access the brain through direct transportation across the blood–brain barrier (BBB). Interestingly, increased BBB permeability is a feature of many neuropathological conditions (reviewed in [26]). Modulating the microbiota may therefore reduce an inflammatory response, improve intestinal barrier function, and prevent proinflammatory cytokines directly accessing the brain. Known pathogens such as *Helicobacter pylori*, *Clostridium perfringens*, *Shigella flexneri*, enterohemorrhagic *Escherichia coli*, and enteropathogenic *E. coli* degrade intestinal barrier function, while organisms like *Faecalibacterium prausnitzii* and *Akkermansia muciniphila* may improve it [27]. Further, selected probiotic strains from the genera *Lactobacillus* (*sensu lato*) and *Bifidobacterium* have been observed to improve barrier function and exert anti-inflammatory effects [28,29].

### 2.4. Enteroendocrine Regulation

The complex interaction between digesta and the small and large intestine induces the release of an array of gastrointestinal hormones from specialized enteroendocrine cells (EECs) distributed in the gut epithelium. These hormones, among others, regulate gastric emptying, intestinal motility, appetite, and postprandial glucose metabolism [30]. Gut microbes can influence appetite and feeding behaviors by modulating the production of such hormones from EECs. 

L-cells, which are embedded mainly in the ileal and colonic epithelium, secrete glucagon-like peptide-1 (GLP-1) in response to nutrients in the small intestine. However, more distally in the intestine they are activated by luminal factors including SCFAs, bile acids, and microbial metabolic products. GLP-1 can interact with the HPA axis and the immune system [31]. In humans, GLP-1 and its receptors have been suggested to reduce anxiety [32]. Glucose-dependent insulinotropic polypeptide (GIP) is released in response to macronutrients from the enteroendocrine K-cells, distributed predominantly in the upper small intestine. GIP has been shown in animal models to reduce anxiety-like behavior [33]. Cholecystokinin (CCK) is secreted in response to the ingestion of macronutrients by enteroendocrine I-cells, located in the duodenum and upper jejunum, and stimulates the release of digestive enzymes and bile. CCK further contributes to reduced appetite [30], and has been observed to increase anxiety-like behavior [32]. Peptide YY (PYY) is co-released with GLP-1 from L-cells. PYY participates in the regulation of appetite and energy intake and has been reported to reduce stress and anxiety responses, and to improve mood [34]. Ghrelin is mainly produced in the gastric mucosa and is involved in the regulation of intestinal motility and appetite [35]. Ghrelin is found in plasma in two major forms: acyl-ghrelin increases appetite and decreases insulin secretion and sensitivity, while des-acyl-ghrelin suppresses appetite and increases insulin secretion and sensitivity [36]. Ghrelin secretion is increased in response to stress; however, chronic stress over time leads to ghrelin resistance and increased secretion of ghrelin, and this has been associated with cravings [37].

### 2.5. Neurotransmitters

Neurotransmission, i.e., the process driving the transfer of information between neurons and their targets, can be influenced by the gut microbiota, which have been shown to produce a range of major neurotransmitters such as dopamine, norepinephrine, serotonin, γ-aminobutyric acid (GABA), nitric oxide (NO), melatonin, histamine, and acetylcholine (Ach). These neurotransmitters provide a possible mechanism of action for how the effects of the gut microbiota on mental and brain health are mediated. The most common neurotransmitters and their function are listed in Table 1.

## 3. The Microbiota–Gut–Brain Axis in Stress and Related Disorders, and Opportunities by Probiotics to Relieve or Prevent Symptoms 

Nutritional psychiatry has developed as a recent field of research given the implication of the microbiota-gut–brain axis in influencing stress-related behaviors, including those relevant to anxiety and depression. A key question is whether targeting the microbiota–gut–brain axis may offer a therapeutic strategy for preventing and/or treating the symptoms of stress-related disorders. To date, several probiotic interventions conducted in healthy participants and psychiatric patients have reported beneficial physiological and psychological effects on several endpoints related to stress and mood. 

### 3.1. Stress, Anxiety and Probiotics

Stress occurs when the normal homeostasis of an organism is disrupted because of an actual or perceived threat and can be categorized as either acute or chronic. Acute stress activates the HPA axis, causing an immediate release in cortisol to respond appropriately to the stressor, which can induce anti-inflammatory responses, thereby preparing the individual for defense against the presented threat. Over time, chronic stress leads to dysregulation of the HPA axis, increasing the risk of consequent side effects, such as mood and stress-related disorders, cancer [38], cardiorespiratory, metabolic, and immune system problems (reviewed in [39]). Today, chronic stress is a rapidly growing global societal challenge [40]. 

Stress can alter the gut–brain axis and has been shown to have a direct impact on the gut microbiota across numerous different animal models, including rodents [41,42,43] and non-human primates [44,45] (also reviewed in [46,47]). Cortisol, the primary stress hormone, has a direct influence on the ENS and vagus nerve, resulting in alterations in the gut microbiota composition [24]. In this field of research, a pioneering preclinical study conducted in germ-free (GF) mice found an exaggerated HPA axis response to stress, which could be normalized by subsequent colonization with *B. infantis* [48]. Acute stress has been shown to influence the microbiota community profile in mice by causing alterations in the relative proportions of the main microbiota phyla [49]. Chronic stress is linked to decreased fecal lactobacilli in rhesus macaques experiencing maternal separation early in life, concomitant with an increase in offspring stress-related behaviors [44]. Furthermore, the transfer of maternal vaginal microbiota from stressed dams to non-stressed pups resulted in an alternation in their response to stress later in life [50]. In humans, infants of mothers with high cumulative stress levels during pregnancy had an altered gut microbiota composition with lower levels of lactobacilli and bifidobacteria, higher levels of potentially pathogenic bacterial taxa, and an increase in maternally reported adverse health symptoms [51]. 

There is growing evidence to suggest that manipulating the gut microbiota through probiotics could modulate stress-related behavior and HPA axis activity [46]. To date, the focus has been on bifidobacteria and lactobacilli, with both preclinical and clinical studies demonstrating promising effects on stress and psychiatric disorders such as anxiety and depression [24,47,52]. In this regard, preclinical models have shown beneficial effects of bifidobacteria and lactobacilli to ameliorate stress-induced behavioral alterations across the lifespan, indicative of a link between the gut microbiota and the stress response. For example, *Companilactobacillus farciminis* prevented the hyperactivation of the HPA axis elicited by acute stress, which the authors hypothesized was a result of the prevention of excessive gut permeability associated with acute stress [53]. Sprague Dawley rats exposed to chronic restraint stress also showed improved anxiety- and depression-like behavior and improved cognitive function following administration of *Lactobacillus helveticus* MCC1848 [54]. *Lactiplantibacillus plantarum* supplementation alleviated heightened stress responses as a result of both chronic unpredictable stress and sleep deprivation stress [55]. Supplementation with *Bifidobacterium* spp. has also been reported to alleviate stress-induced behavioral alterations in preclinical models [56,57]. 

Moving from preclinical to clinical evidence, probiotics have been proven to have some success in ameliorating mood in a number of clinical studies [58,59]. Improvements in mood scores were observed in elderly participants following administration with a milk drink containing *Lacticaseibacillus casei*, proving most beneficial in the participants that reported the lowest mood scores at baseline [60]. A multi-species combination of *Streptococcus thermophilus*, *Lactobacillus delbrueckii* subsp. *bulgaricus*, *Lactococcus lactis*, *Lactobacillus acidophilus*, *L. plantarum*, *Bifidobacterium animalis* subsp. *lactis* and *Limosilactobacillus reuteri* administered to healthy participants elicited anxiolytic effects [61], whereas another multi-species combination (nine strains, including *Lactobacillus* (*sensu lato*), *Lactococcus*, and *Bifidobacterium*) ameliorated cognitive reactivity to sad mood in healthy participants [62]. Of note, one later study using the same combination demonstrated that the neurocognitive benefits of this multi-species probiotic became evident only when the participants were stressed, highlighting the need to carefully characterize study populations [63]. A multi-species combination (containing *L. fermentum* LF16, *Lacticaseibacillus rhamnosus* LR06, *L. plantarum* LP01, and *B. longum* BL04) induced significant improvements in mood, with a reduction in depressive mood state, anger, and fatigue, and an improvement in sleep quality in healthy volunteers [64]. *L. plantarum* DR7 administration to stressed adults alleviated stress and anxiety, as well as improving several aspects of memory and cognition, enhanced serotonergic signaling, and decreased plasma cortisol and proinflammatory cytokines [65]. Intake of *L. plantarum* HEAL9 also led to a significant decrease in the plasma levels of two inflammatory markers (soluble fractalkine and CD163) following exposure to an acute stress test [66]. A 12-week intervention with *Lactobacillus gasseri* and *B. longum* also resulted in positive changes in stress and salivary cortisol measurements and concomitant improvements in immune response in healthy participants [67]. Healthy medical students undergoing university examinations had reduced levels of stress following the consumption of a fermented milk containing the probiotic *L. casei* Shirota [68]. Furthermore, increases in salivary cortisol reported during an exam stress period were also reduced in a healthy student population supplemented with *L. plantarum* 299v [69]. A multi-species probiotic administered to healthy college students was found to improve panic anxiety, neurophysiological anxiety, negative affect, worry, and increase negative mood regulation [70]. Further, supplementation with *L. casei* [71] and *Bifidobacterium bifidum* [72] reduced the physical symptoms of exam stress, including the onset of stress-induced gastrointestinal symptoms and head colds. Finally, an open-label study conducted in highly stressed information technology specialists found that administration of *L. plantarum* PS128 improved several self-reported and objective measures of mood, anxiety, stress, and sleep [73]. 

Taken together, these examples highlight significant results and describe an intriguing role of probiotics in mood, anxiety, stress, and related behaviors such as sleep. A recent meta-analysis demonstrated that probiotic consumption could result in a reduction of subjective stress levels in healthy volunteers and may alleviate stress-related subthreshold anxiety and depression levels [58]. However, further clinical studies are required to provide a deeper understanding of the strain specificity and mechanisms of action of probiotics to help fully realize their role in stress management and the relief of the symptoms of anxiety.

### 3.2. Depression and Probiotics

Major Depressive Disorder (MDD) is a common psychiatric disorder characterized by depressed mood or significantly reduced pleasure or interest in all activities and is currently a leading cause of disability worldwide. Emerging evidence shows that the dysfunction of the gut–brain axis may be implicated in the etiology of depression. In support of this, the gut microbiota are impacted by MDD and associated with changes to gut epithelial permeability and increased systemic inflammation with elevated levels of C-reactive protein, IL-1β, IL-6, and TNFα in depressed patients compared with healthy controls [74]. Furthermore, the “leaky gut” phenomenon resulting from disrupted gut barrier function is proposed to contribute to MDD. In this context, MDD patients show elevated serum concentrations of immunoglobulin (Ig)-M and IgA against lipopolysaccharides of Gram-negative bacteria compared to healthy controls [75], suggesting an increase in bacterial translocation from the gut and subsequent inflammatory response, potentially contributing to an MDD phenotype. 

Preclinical models of depression, such as the maternal separation model [76] and the Flinders-sensitive rat model [77], have demonstrated alterations in the gut microbiota composition and inflammation. In humans, many studies have examined alterations in the gut microbiota in MDD patients compared to healthy controls [78,79,80,81]. Specifically, MDD patients are reported to have an altered gut microbial compositional profile relative to healthy controls [80,81,82]. Patients with MDD have been reported to have reduced abundances of Bacteroidetes, Firmicutes, and Actinobacteria with a concomitant outgrowth of Proteobacteria [81,82], and increased abundance levels of *Alistipes* spp. [80]. The Flemish Gut Flora project provided further associations between the gut microbiota profile in a depressive cohort by highlighting the absence of *Coprococcus* and *Dialister* species in patients with depression [83]. However, several variations have been reported across these studies, which may be due to the small sample sizes or the effects of adjunct medications [47]. 

The use of probiotics for the reduction of symptom severity in MDD has gained attention in recent years, indicated by increasing numbers of preclinical and clinical studies that have supported the anti-depressive efficacy of probiotics. Accumulating preclinical evidence indicates that single-strain or multi-species preparations may be effective in improving the behaviors related to depression (reviewed in [47]). In a recent study, a multi-species probiotic combination of *L. plantarum* LP3, *L. rhamnosus* LR5, *B. lactis* BL3, *B. breve* BR3 and *Pediococcus pentosaceus* PP1 alleviated depressive-like behaviors and decreased corticosterone levels in mice subjected to restraint stress [84]. Similarly, *L. plantarum* WLPL04 alleviated anxiety- and depressive-like behaviors and chronic stress-induced cognitive dysfunction in mice, while also reversing abnormal alterations in the composition of the gut microbiota [85]. 

In humans, administration of *B. longum* NCC3001 for six weeks to adults with irritable bowel syndrome (IBS) and mild to moderate anxiety and/or depression reduced depression scores and enhanced the participants’ quality of life, which was associated with alterations in brain activation patterns in the limbic system. No improvement in anxiety scores were observed in this cohort [86]. Another study investigated the effect of *B. coagulans* MTCC 5856 in patients experiencing co-morbid IBS symptoms with MDD and found that the probiotic significantly improved symptoms of both depression and IBS [87]. Slykerman and colleagues found that *L. rhamnosus* HN001 supplementation during pregnancy resulted in a significant reduction of postnatal depression and anxiety symptoms [88]. An improvement in cognition was reported in a cohort of depressed patients receiving *L. plantarum* 299v compared to the placebo group [89]. MDD patients who were administered *L. acidophilus*, *L. casei*, and *B. bifidum* for eight weeks also reported ameliorations in self-reported depression scores [90]. Finally, an open-label study conducted in patients with treatment-resistant depression highlighted the potential of probiotics as an adjunct therapy with antidepressant drugs [91]. 

Further evidence to support these clinical findings are reported in several systemic reviews [92,93,94]. However, it is important to note that several intervention studies failed to demonstrate any beneficial effects on improving overall mood (reviewed in [95,96,97]). It is evident from the preclinical research that specific bacterial strains play a role in ameliorating depressive-like behaviors, and certain clinical studies have demonstrated a role for probiotics towards alleviating symptoms of depression; however, the exact bacterial species and/or strains and mechanisms underpinning their beneficial effects remain unclear. Nevertheless, current research highlights the importance of a healthy microbiome for patients suffering from depression. Future studies into the strain-dependent nature of putative probiotics in patients with clinically diagnosed MDD are warranted to evaluate their therapeutic potential.

## 4. From Preclinical to Clinical—Translational Challenges in Microbiota–Gut–Brain Axis Studies 

**Figure 1:**
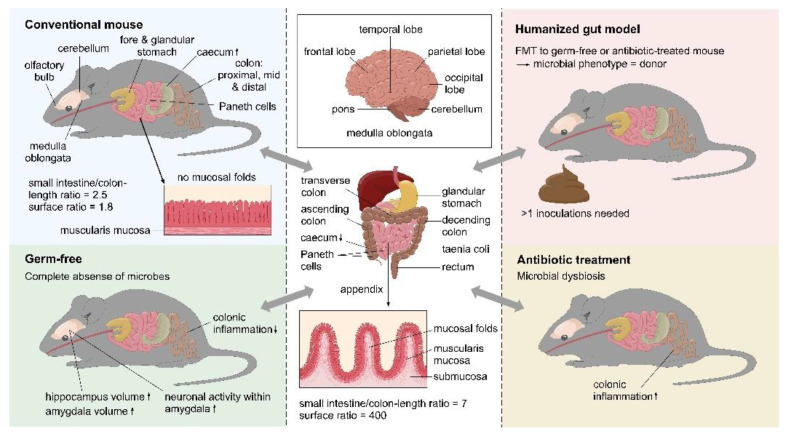
Physiological comparison of the brain and gastrointestinal tract of mice and humans. The differences between a conventional mouse, a germ-free mouse, a humanized mouse, and an antibiotic treated mouse are also shown. The ↑ indicates increased size, while ↓ indicates decreased size. ©Pinja Kettunen/SciArt & IFF, with permission.

Most of the evidence related to the pathways of communication along the microbiota–gut–brain axis, the proposed mechanisms of targeting the microbiota to influence brain function and behavior, and the role of host–microbiota interactions in stress and related disorders is derived from preclinical studies, predominantly using rodent models. However, while animal models have unquestionably been proven invaluable in enabling researchers across various scientific and medical fields to narrow the gaps in our understanding of the microbiota–gut–brain axis, the translation of these findings from mouse to man has proven difficult, particularly from rodent models where so many confounding factors can be controlled. Figure 1 describes the physiological differences between mouse and human, while the changes and effects with the key models are listed in Table 2. Although these models each have their specific deficits, they have proven tantamount for accumulating evidence for the microbiota–gut–brain axis, but have translatability challenges, further focusing on specific case studies of probiotic intervention for the translation of results from preclinical models to clinical populations. 

### 4.1. Germ-Free Models 

GF animals are those lacking any microbial exposure since birth, and although extremely abnormal, research in GF mice has significantly enhanced our understanding of the crucial role of microorganisms across virtually all physiological processes in the host [98]. GF mice have revealed the importance of the microbiota for normal aging, immune-, metabolic-, digestive-, and gastrointestinal function, and the normal development and function of the nervous system [99]. For an extensive overview of the impact of the complete absence of gut microbiota on brain physiology, function, and behavior of the GF mouse, please see [98]. 

Sudo and colleagues were the first to demonstrate that the exposure of GF mice to an acute experimental stressor resulted in an exaggerated increase in glucocorticoid production from the HPA axis compared with SPF control mice [48]. Interestingly, monocolonization with either *B. infantis* or enteropathogenic *E. coli* in early life could either normalize or further exacerbate the HPA axis response to stress observed in GF mice, respectively [48]. This effect of the GF condition on stress responsivity has since been reproduced in a different mouse strains [100] and in rats [101].

Research in GF models has enhanced our knowledge of the microbiota–gut–brain axis and has provided the most convincing evidence towards elucidating the pathways of microbial impact on the development and function of several key physiological systems, in this case the impact on the nervous system. These important discoveries in GF models can be used to guide research and innovation aimed at developing new therapeutic solutions that target the gut microbiota and impact brain health, but since the GF condition has such far reaching consequences on the body as a system, the translatability to the clinical condition is very limited.

### 4.2. Antibiotic Models 

Unquestionably, antibiotics are one of the most important influencing factors on the gut microbiome, and in addition to GF models, antibiotic models have provided us with another useful tool for investigating the impact of the gut microbiome on health. Antibiotic models enable more specific targeted disruption of the gut microbiota as opposed to the complete absence of gut microbiota in the GF model. In the context of translation, antibiotic models can be tailored to resemble the clinical scenario in humans more closely. For example, the extent to which the microbiota are depleted, the dose of antibiotics used, the duration of treatment, the composition of antibiotic used (single or cocktail), and the timing of life during which the antibiotics are administered can all be controlled to mimic various clinical scenarios [47]. Such methodological tweaks enable researchers to determine the effect of antibiotic-associated gut microbiome disruptions on brain functions and behavior. 

The collective studies using antibiotic models (Table 2) provide significant evidence that gut microbiota depletion during critical windows of development—potentially already starting at the periconception period, but certainly into adolescence—significantly impacts brain function and behavior throughout life. Antibiotic administration also has an impact on immune, behavioral, and neurochemical markers in the brain [102,103,104,105,106,107]. Irrespective of the methods used for microbiota depletion, these findings have potentially significant translational relevance considering the clinical use of antibiotics in both mothers and infants. Although these results are intriguing, and certainly describe a role of the gut microbiome in neurodevelopment in early life, symptom onset, and the progression of brain disorders, it must be acknowledged that the clinical setting in all cases is much more complex and thus, the translation of these findings is complicated. 

### 4.3. Humanized (Gut) Models

In the context of the microbiota–gut–brain axis and mechanistic studies to underpin the role of the gut microbiome in brain health, animals, most commonly mice, can be humanized following engraftment with parts of the human gut microbiota. This is done through fecal microbiota transplant (FMT), the process of transferring the gut microbiota from humans into either a GF mouse or a mouse pretreated with antibiotics to deplete the murine gut microbiota. The subsequent repopulation of the mouse gastrointestinal tract with the microorganisms transplanted from the human fecal matter results in the generation of a humanized mouse with a gut microbiota resembling that of the human donor [9]. The translational challenge is, of course, that the gut microbiome is the only humanized component of the rodent, and the background of the recipient mouse should also be seriously considered. Prior to the FMT, GF recipient mice are already markedly altered and depending on the dose, duration, and composition of the antibiotic cocktail, antibiotic-treated recipient mice may have experienced other CNS effects or incomplete microbiome depletion. Settanni et al. recently reviewed the evidence on FMT for brain health and extensively documented the studies in which FMT has been used to induce psychiatric symptoms in rodent models [108]. The gut microbiota from donors with depression [81,82], alcoholism [109,110], anorexia nervosa [111], IBS [112], and schizophrenia [113,114] have all been transplanted into rodents that later presented abnormalities in behavior, indicating at least partial transfer of the clinical psychiatric phenotype. Although not all of the phenotypic characteristics of patients with depressive disorder were transplanted via the microbiota, and the number of donors in both studies was low [81,82], these models do provide some opportunity to investigate microbiome-associated depression and novel microbiome-targeted therapeutics such as probiotics and custom diets. One major challenge with FMT studies is the engraftment of the donor microbiota in the host; thus, booster inoculations are often administered to curtail this [81]. 

Outside of humanized rodent models, FMT has been used most frequently and successfully in the clinical setting to treat recurrent *Clostridiodes difficile* infection [115] and has been investigated in a limited capacity to treat psychiatric disorders (extensively reviewed in [108]). In addition, FMT has also been performed within species to demonstrate the transplantation of an anxious phenotype from a mouse strain (BALB/c), that displays a more innately anxiety-like behavior, to a GF model of a mouse strain (NIH Swiss), that displays less anxiety-like behavior, thus further implicating the microbiome in anxiety [116]. FMT has also been used to demonstrate the impact of prenatal stress on the maternal microbiome, maternal-to-offspring microbiota transmission, and associated stress-related deficits on the offspring’s brain and gut [50].

Overall, humanized (gut) rodent models have led to several breakthrough discoveries and have significantly enhanced our understanding of the role of the gut microbiome in brain health. Although the translation to the clinical setting is challenging, these models should be considered within preclinical study design as a crucial step towards bridging the gap between mouse and man. 

**Table 2:** The alterations of the key models affecting brain physiology and function as well as behavioral profiles, cognitive function, and stress responses in mice.

Model		Changes	Effects		References
Germ-free	Brain physiology and function	Increase of neurogenesis in adult GF mice	Important role in learning and memory		[117]
		Increased hippocampal and amygdalar volume, altered dendrite and neuronal morphology within these brain regions	Structural integrity and signaling pathways within brain regions involved in stress response, anxiety behavior, and social interactions are dependent on the presence of the gut microbiota		[118,119]
		Increased neuronal activity within the amygdala is associated with upregulated genes	GF mice have significantly lower BDNF mRNA expression compared to specific pathogen-free (SPF) mice		[119]
		Lack of gut microbiota	Significant effect on serotonergic neurotransmission within CNS		[100]
		Hippocampal concentrations of serotonin and 5-hydroxyindoleacetic acid, 5-HIAA (main metabolite of serotonin) are increased in male GF mice, and plasma concentrations of tryptophan are also increased			[100]
		Decreased expression of the serotonin receptor 1A (5HT1A) in the hippocampus			[120]
		Both increase and decrease of hippocampal BDNF mRNA expression reported in different studies			[100,120,121]
		Upregulation of genes linked to myelination and myelin plasticity in prefrontal cortex of adult GF mice	Presence of hyper-myelinated axons within prefrontal cortex	Significant impact on the future development of treatment strategies for myelination diseases, such as multiple sclerosis	[122]
		Absence of gut microbiota	Microglia of GF mice are defective and display an immature phenotype and an impaired innate immune response to infection with a bacterial-associated inflammatory mediator—lipopolysaccharide (LPS)	The immune response is also defective within the periphery	[24,100,123]
		Increase in BBB permeability	The CNS of GF mice is particularly vulnerable to brain damage and infection		[124]
	Behavioral profiles, cognitive function and stress responses	Absence of gut microbiota	Increased pain response and visceral sensitivity		[125,126,127,128]
			Impairment in sociability and social cognition, although one study has shown the opposite	i.e., the gut microbiome is essential for normal social behavior	[126,127,128,129]
			Impaired short-term recognition and working memory		[130]
		Hyperactivity of the HPA axis response to stress	Varied effect of anxiety-like behavior, depending on the experimental design, species, strain, and sex		[100,101,116,120,121,126,130,131]
Antibiotic	During critical windows	Alterations or depletion of microbiota through administration of antibiotics (single/cocktail, absorbable/non-absorbable) to dams either during the periconception period, and/or during pregnancy, and/or during weaning or to the offspring in early life	Effect on neurodevelopment and behavior		[132,133,134,135]
			Reduced anxiety-like behavior Increased aggressive behavior Increased resilience to stress Reduction of social behavior and preference for social novelty Altered cytokine expression in the brain Altered BBB integrity		[136]
			Increased visceral hypersensitivity		[137,138]
			Reduced anxiety-like behavior, cognitive deficits, altered tryptophan metabolism and gene expression		[139]
			Expression of BDNF and its receptor in both ENS and CNS		[140]
			Expression of genes involved in immune function, neurotransmission, and neuroplasticity in the amygdala	Long-lasting effects on gut microbiota composition into adulthood	[141]
	In mood disorders and neurodegenerative disorders	Alterations in gut microbiota	Attenuated inflammation, and β-amyloid (Aβ) and other pathologies associated with disease progression Delay disease related memory deficits		[142,143,144,145]
		Depletion of gut microbiota	Decreased microglia activation, reduced	Indication that gut microbiome is important for enhancing Parkinson’s disease-like symptoms	[146]
		Administration of an antibiotic cocktail from adolescence to adulthood to mice with experimental autoimmune encephalomyelitis (EAE)t	Depletion of the gut microbiota significantly delayed the onset of EAE symptoms and altered several immunological and neurobehavioral responses		[147]
		Administration of antibiotics prior to exposure to chronic social defeat stress (CSDS)	No development of anhedonic-like behavior in adulthood when compared to mice administered water only		[148]
		Administration of antibiotic cocktail	Depleted serotonin levels in the intestine coupled with an altered sleep/wake cycle		[149]
Humanized gut models		Generation of a humanized mouse with a gut microbiota resembling that of the human donor	GF recipient mice are already markedly altered and depending on the dose, duration, and composition of the antibiotic cocktail, antibiotic-treated recipient mice may have experienced other CNS effects or incomplete microbiome depletion	FTM from human donors with, e.g., depression, alcoholism, anorexia nervosa, IBS, and schizophrenia; rodents later presented abnormalities in behavior, indicating at least partial transfer of the clinical psychiatric phenotype	[81,82,109,110,111,112,113,114]
		Transplantation of the gut microbiota from patients with PD	Worsening of the motor symptoms in genetically susceptible mice compared with those in receipt of the gut microbiota from healthy controls	Highlight the gut microbiome as a significant contributing factor towards progression of PD symptoms in genetically susceptible hosts	[146]
			Recipient rats developed behavioral and physiological features characteristic of the donors with depressive disorder such as increased anhedonic- and anxiety-like behaviors, as well as alterations in tryptophan metabolism and an increased inflammatory profile		[113,114]

## 5. Psychobiotics—Selected Case Studies of Translational Results from Preclinical to Clinical

Recent evidence in the literature suggests a psychobiotic potential for selected probiotic strains and strain combinations. Here, four of such strain combinations will be discussed in more detail.

### 5.1. Bifidobacterium longum 1714^®^

*B. longum* 1714^®^ has been investigated across several preclinical and clinical trials with successful translational results. Here, we will review the psychobiotic evidence for this strain, starting with preclinical models and then moving towards clinical populations. 

Innately anxious but otherwise healthy BALB/c mice were administered *B. longum* 1714 at a dose of 1 × 10^9^ colony forming units (CFU) per day, or a commonly prescribed selective serotonin reuptake inhibitor and compared to a vehicle group. While *B. longum* 1714 and escitalopram reduced compulsive and anxiety-like behavior in the marble burying test, subsequent behavioral tests showed that the strain further reduced stress- and depression-related behavior more efficiently than the antidepressant drug [57]. In a follow-up study, improvements in several aspects of cognitive function were demonstrated in BALB/c mice that were administered *B. longum* 1714, at the same dose as previously described, compared to a vehicle group [150]. The authors concluded that the impact of *B. longum* 1714 on cognitive function could be associated with reduced anxiety. Taken together, these preclinical findings highlighted *B. longum* 1714 as a candidate psychobiotic worthy of clinical investigation. 

In the first clinical trial, healthy volunteers were recruited for a within-participants, repeated measures, placebo-controlled clinical trial to investigate the effects of *B. longum* 1714 on the stress response, cognition, and brain activity patterns [151]. At baseline, the participants completed the socially evaluated cold pressor test (SECPT) coupled with the state portion of the state-trait anxiety inventory to measure the combined psychological and physiological response to an acute stress, and a resting electroencephalography (EEG) recording was taken to measure brain activity prior to cognitive assessments. The participants received a placebo for the first four weeks, followed by a four-week intervention of *B. longum* 1714 at a daily dose of 1 × 10^9^ CFU. Further, the study had a two-week follow-up after probiotic administration. *B. longum* 1714 reduced cortisol output during the SECPT compared to placebo and the baseline assessment. Subjective anxiety was significantly increased in response to the acute stress at baseline and following intervention with placebo; however, this was attenuated following intervention with *B. longum* 1714. Perceived stress was marginally reduced following the *B. longum* 1714 intervention compared to placebo, and perceived stress levels also increased in the two-week follow-up post *B. longum* 1714 intervention. Finally, *B. longum* 1714 improved visuospatial memory performance that was coupled with an EEG profile consistent with improved memory. These results indicated the early psychobiotic potential of *B. longum* 1714, consistent with the preclinical findings [151]. A subsequent randomized, double-blind, placebo-controlled clinical trial involved healthy adults allocated to receive either a placebo or the same dose of *B. longum* 1714 for four weeks [152]. Brain activity was measured using magnetoencephalography in response to social stress characterized by social exclusion and rejection induced by a standardized social stress paradigm. In addition, the participants health status was measured at baseline and at the end of the study using a self-report questionnaire. Although there was no effect overall on health status, *B. longum* 1714 modulated resting state neural activity, which correlated with enhanced energy/vitality [152]. Furthermore, all participants experienced an increase in social stress in response to the social stress paradigm, but *B. longum* 1714 altered the neural responses during social stress—brain activity which may play a role in the activation of brain-coping centers to counter-regulate negative emotions [152]. However, Moloney et al. [153] conducted a randomized, placebo-controlled, repeated measures study where healthy students consumed both placebo and *B. longum* 1714 for eight weeks in a crossover design during their preparation for university semester examinations. The post-intervention assessments took place during the examination period, a naturalistic chronic stressor in this study. Self-reported stress, anxiety and depression, and cortisol output only marginally increased from a very low baseline level in response to exam stress, and *B. longum* 1714 did not alleviate any of these symptoms [153]. A positive effect of *B. longum* 1714 compared to placebo on sleep duration was observed when analyzing the change from baseline scores [153]. Given the differences in the model of prolonged chronic stress in preparation for university semester examinations and the acute stress tests that were applied in the previous studies, it could be hypothesized that candidate psychobiotics could be more effective in participants with moderate anxiety or under exposure to an artificial stressor. 

### 5.2. Lacticaseibacillus paracasei Lpc-37^®^

*L. paracasei* Lpc-37^®^ has been investigated across two preclinical studies using the same chronic stress model and in one clinical trial with healthy participants. The translation of the results from preclinical to clinical will be discussed here.

Chronically stressed mice were administered one of twelve candidate strains at a daily dose of 1 × 10^9^ CFU for a total of five weeks, after which behavior and the neuroendocrine response to stress were investigated. Of the twelve strains, *L. paracasei* Lpc-37, *L. plantarum* LP12407, and *L. plantarum* LP12418 were most effective at attenuating anxiety- and depression-related behavior following chronic stress, and this was observed across two independent experiments in the same model [154]. A reduction in corticosterone was observed following intervention with *L. paracasei* Lpc-37 [154]. Crucially, there were eight candidate probiotic strains included in this large screening experiment that had no effect on anxiety- and depression-related behavior, highlighting the challenges in discovering a candidate psychobiotic, even in a model where mice display anxiety-like behavior and where environmental factors such as diet are tightly controlled. 

A randomized, double-blind, placebo-controlled clinical trial was established whereby healthy participants (*n* = 118) received either placebo or *L. paracasei* Lpc-37 at 1.75 × 10^10^ CFU per day for five weeks [155]. It was observed that self-reported perceived stress was significantly reduced with *L. paracasei* Lpc-37 compared to the placebo group [155]. *L. paracasei* Lpc-37 was observed to reduce the increase in heart rate in response to the acute stress in participants with low chronic stress; the opposite was observed in participants with high stress levels. Such a result could suggest that the effect of *L. paracasei* Lpc-37 on ANS response to stress based on heart rate may be differentially dependent on chronic stress. Further significant effects were identified within the subgroups, where it was shown that *L. paracasei* Lpc-37 increased perceived productivity, feelings of restfulness after a night’s sleep, and perceived health and reduced diastolic blood pressure in participants with high stress levels compared to the placebo group. In participants with low stress levels, *L. paracasei* Lpc-37 reduced fatigue levels during the acute stress procedure and normalized evening cortisol levels. Furthermore, *L. paracasei* Lpc-37 was also shown to significantly reduce perceived stress in females following the five-week intervention in comparison to the placebo group, whereby females could be considered a more stress-vulnerable population in this study [155]. Taken together, the results of these studies highlight *L. paracasei* Lpc-37 as an interesting psychobiotic candidate with limited, but consistent, translation of stress-reducing results from a preclinical model to a clinical trial. 

### 5.3. Lactobacillus helveticus Rosell^®^-52 + Bifidobacterium longum Rosell^®^-175

The combination of *L. helveticus* Rosell^®^-52 (R0052) and *B. longum* Rosell^®^-175 (R0175) has been investigated across various preclinical models and clinical trials of healthy and patient populations for several years, with some translational findings, but other conflicting results. 

The combination of *L. helveticus* R0052 and *B. longum* R0175 was first investigated in different experiments of myocardial infarction (MI) [156,157,158]. It was observed that administration of the probiotic combination for just seven days prior to inducing a MI, and then again for ten days beginning from the seventh day post-MI until euthanasia, prevented MI-induced depression-like behavior and deficits in social interaction from developing and restored intestinal barrier integrity in MI rats [156]. A subsequent study showed that the probiotic combination reduced pro-apoptotic activity within specific brain regions, and attenuated deficits in social interaction behavior and depression-like behavior [157]. Later, it was discovered that the beneficial effects of the probiotic combination on preventing the symptoms of MI developing were dependent on the integrity of the vagus nerve [158].

Other early investigations demonstrated that a two-week administration of the probiotic combination reduced anxiety-like behavior in rats [159]. In a mouse model of chronic psychological stress, a significant reduction in stress-induced increases in plasma corticosterone and catecholamines was noted, and modulated specific markers of neuronal activity and attenuated stress-induced alterations in hypothalamic synaptic-plasticity-related neuronal networks, stress-induced deficits in hippocampal neurogenesis, and stress-induced intestinal permeability were observed [160]. In a follow-up experiment using the same design and model, the probiotic combination was shown to attenuate chronic stress-induced visceral hypersensitivity and increases in plasma corticosterone and catecholamines. Moreover, the probiotic combination also prevented the stress-induced reduction in the mRNA expression of the glucocorticoid receptor in several brain regions associated with the stress response. [161]. In a model of LPS-induced peripheral and neuroinflammation, two weeks of pretreatment with the probiotic combination reduced the circulating and protein expression levels of pro-inflammatory cytokines in the hippocampus [162]. Although systemic exposure to LPS induced deficits to cognitive function, the probiotic combination demonstrated no effect at the behavioral level but did prevent LPS-induced deficits in BDNF expression levels in the hippocampus [162]. In a genetic rodent model of depression, the probiotic combination reduced the depression-associated increase in noradrenaline levels in the plasma and reduced the increase in dopamine levels in the plasma. Neither dose of the probiotic combination had any effect on brain monoamine levels or cognition and anxiety- or depression-like behaviors [163]. In a hamster model of social defeat stress, the probiotic combination significantly increased social avoidance and decreased social interaction in hamsters who displayed anxiety-like behavior, whereby this increase in social anxiety-like behavior was associated with reduced microbial diversity following social defeat [164]. This behavioral result somewhat conflicts with previous studies, which reported a reduction in anxiety-like behavior following intervention with the probiotic combination. 

The potential psychobiotic effect of *L. helveticus* R0052 and *B. longum* R0175 in combination has also been investigated in clinical trials. Healthy adults affected by self-reported daily stress reported significantly reduced abdominal pain and nausea/vomiting compared to the placebo group [165]. There was no effect of the probiotic combination on any of the other gastrointestinal symptoms of stress that were assessed or on any of the other physical or psychological discomforts induced by stress [165]. Healthy but borderline clinically anxious and depressed adults consuming the probiotic combination reported a greater reduction in the median global symptom severity index percentage change scores following intervention compared to the placebo group. This result was attributed to greater reductions in the percentage change scores for somatization, depression, and anger–hostility. Furthermore, the median percentage change for the global Hospital Anxiety and Depression Scale (HADS) score in participants who were supplemented with the probiotic combination was reduced more than that of the placebo group from baseline to the end of the study. However, it should be noted that the median HADS global score was exactly the same within both groups at the end of the study [159]. In a post hoc analysis conducted on “less stressed” participants, the percentage change scores for perceived stress and obsessive–compulsive, anxiety, and paranoid ideation were further reduced significantly following supplementation with the probiotic combination compared to placebo [166]. 

Depressive patients free of any psychiatric medication were recruited for the next randomized, double-blind, placebo-controlled trial to investigate the effects of 3 × 10^9^ CFU per day of *L. helveticus* R0052 and *B. longum* R0175 for eight weeks. There was no significant effect of the probiotic combination on any of the nine psychological outcome measures included in the study, or on any blood-based biomarker (CRP, IL-1β, IL-6, TNF-α, and BDNF) [97]. However, in the probiotic group, it was observed that participants with high levels of vitamin D at baseline experienced a significantly greater improvement in several psychological outcomes over time compared to those with low baseline levels of vitamin D [97]. The probiotic combination was next investigated in patients with MDD who were taking antidepressant drugs for three months or more before the trial commenced. The combination of *L. helveticus* R0052 and *B. longum* R0175 significantly reduced depressive symptoms as measured using the Beck Depression Inventory (BDI), compared to the placebo group [167]. Lastly, the probiotic combination was investigated in treatment-naïve patients with MDD. In both studies, the effect of the probiotic combination at the dose of 3 × 10^9^ CFU per day for eight weeks was investigated in 10 patients per study with a score of ≥20 in the Montgomery Åsberg Depression Rating Scale indicating at least moderate depression. The studies showed a significant improvement in subjective psychological outcomes, but there was no effect on objective measures of sleep quality [168,169]. It is, of course, unclear to what extent these outcomes are influenced by a placebo effect.

There are already substantial data concerning the psychobiotic potential of *L. helveticus* R0052 and *B. longum* R0175. Based on the evidence discussed above, *L. helveticus* R0052 and *B. longum* R0175 have demonstrated some conflicting results in both preclinical and clinical trials, and the psychobiotic potential of this probiotic formulation is worth investigating further in future clinical trials. 

### 5.4. Lacticaseibacillus rhamnosus JB-1™

*L. rhamnosus* JB-1™ (previously referred to as *L. reuteri*) has comprehensive preclinical evidence that repeatedly demonstrates behavioral, physiological, and neurobiological efficacy. Despite this, *L. rhamnosus* JB-1 has only been investigated in one clinical trial to date, which reported an unsuccessful translation of results. 

It was first shown that *L. rhamnosus* JB-1 inhibited the cardio–autonomic response to colorectal distension (CRD) and the perception of visceral pain evoked by CRD [170]. This discovery led to a hypothesis that *L. rhamnosus* JB-1 could be used as a potential treatment for patients with functional bowel disorders, such as IBS, experiencing abdominal discomfort and pain. It was later shown that the nociceptive effects of *L. rhamnosus* JB-1 resulted from a reduction in CRD-induced dorsal root ganglia excitability [171], and that the strain targets an ion channel in enteric sensory nerves to influence gut motility and pain perception [172]. In addition, the luminal application of *L. rhamnosus* JB-1 to naïve mouse jejunum and colon tissue samples affected gut motility in ex vivo perfusion models [173,174]. In a similar ex vivo model, this time using naïve tissue samples taken from mice previously exposed to an acutely stressful procedure, it was shown that *L. rhamnosus* JB-1 countered the effect of stress-induced dysmotility in both jejunal and colon segments [175]. Taken together, these results point to the ENS and spinal pathways as a potential pathway for gut–brain communication by *L. rhamnosus* JB-1. 

*L. rhamnosus* JB-1 reduced stress-induced corticosterone and anxiety- and depression-like behavior in BALB/c mice innately displaying anxiety-like behavior. This effect on the stress response and behavior was coupled with a reduced GABA_B1β_ receptor expression in the hippocampus and amygdala, which is consistent with the anti-depressant like effect of GABA_B_ receptor antagonists [176]. Remarkably, it was demonstrated that these neurochemical and behavioral effects were not detected in vagotomized mice, thus identifying the vagus nerve as an essential route of gut–brain communication through which *L. rhamnosus* JB-1 mediated effects on brain function and behavior [176,177]. In a separate experiment, magnetic resonance spectroscopy (MRS) later confirmed that intervention with the same dose of *L. rhamnosus* JB-1, also in BALB/c mice, increased levels of three biomarkers of brain function, including glutamate/glutamine and GABA [178]. In a model of chronic psychosocial stress, *L. rhamnosus* JB-1 attenuated stress-induced anxiety-like behavior and deficits in social interaction [179,180,181], coupled with immuno-regulatory action and a dampening of the stress-induced immune response [179], a normalization of serum kynurenine and kynurenic acid, and a restoration of stress-induced abnormalities in tryptophan-kynurenine metabolism [180]. This was independent of any preventative effects on stress-induced dysbiosis [179]. However, the administration of *L. rhamnosus* JB-1 after the social defeat stress perpetuated, rather than prevented, the behavioral and physiological deficits associated with stress exposure [182]. A similar result was demonstrated for the selective serotonin reuptake inhibitor (SSRI) sertraline, indicating that the timing of the intervention is critically important. 

Early life concurrent administration of *L. rhamnosus* JB-1 to antibiotic-treated pregnant dams until weaning decreased anxiety-like behavior in female offspring, and prevented antibiotic-associated deficits in sociability [136]. Administering *L. rhamnosus* JB-1 to antibiotic-treated offspring one week prior to weaning prevented the antibiotic-associated effects, such as deficits in social behavior (but not on anxiety-like behavior), changes in gene expression in specific brain regions, and changes in immune cell populations in the spleen [183]. 

On a general level, when comparing the different mouse models, it appears that BALB/c mice [176] are more responsive compared to C57BL/6 [179] and Swiss Webster mice [184]. BALB/c mice are often preferentially selected as animal models of anxiety and depression, as they better reflect the core features observed in these patient populations [184]. When there are differences between mice strains, it is obvious that translation to humans is even more prone to challenges.

Healthy male participants received *L. rhamnosus* JB-1 for eight weeks and were exposed to an acute stressor; the influence on cognitive function, anxiety, mood, host inflammatory profile, and brain activity patterns measured using EEG were determined [185]. *L. rhamnosus* JB-1 was not found to have an effect on mood, anxiety, stress, sleep quality, memory performance, attention, or anti-inflammatory cytokine levels compared with the placebo. Nor was there any effect on either the psychological response or the neuroendocrine response to the acute stress [185]. In the preclinical studies, *L. rhamnosus* JB-1 only demonstrated beneficial effects on behavior, physiology, and brain function in either stress-sensitive mice or in models of chronic stress. This is very different compared to the healthy human participants that made up the population in this study [185].

This study highlights the fundamental challenges in translating the findings from putative psychobiotic candidates in stress-susceptible animals to healthy human populations. Nevertheless, the preclinical evidence on the psychobiotic potential of *L. rhamnosus* JB-1 is substantial. However, it may be advised that future clinical trials focus on more clinically anxious populations, or in patients with mood disorders such as anxiety and depression. 

## 6. Future Perspectives and Conclusions 

The preclinical animal models enable studies related to the gut microbiota to be conducted in a controlled experimental setup, helping to evaluate the host–microbiota interactions as well as developing mechanistic hypotheses. These have advantages such as a short life cycle and high reproductive rates; however, although the organs in the gastrointestinal tract in a mouse and a human are similar, there are differences in the anatomy. In addition, although many common bacterial genera are found both in the human and mouse intestine, there are large differences, and only about 4% of the bacterial genes are found to share identity. These differences make the translation of gut microbiota research from mouse models to humans challenging. 

Moreover, there is the impact of variability in human clinical trials (i.e., intra- and inter-individual response to dietary supplements) and the challenges that come with assessing the true effect of nutrient bioactives/probiotics in humans within a “non-controlled”/free-living environment, which is not an issue in animal studies. This can be partly managed by reducing the variability of the subject cohort when designing nutritional interventions.

Furthermore, the probiotic dose used in mouse vs. human trials is vastly different. The established formulas for translating the dosage for a mouse into equivalent human doses do not consider the dosage of probiotic foods or supplements, where the dose is based on the number of live organisms present. In addition, differences in the timing and length of probiotic intake may, in part, explain the differences in outcomes.

Fecal samples are frequently used as a proxy of the gut microbiota due to the noninvasive and easy manner of obtaining them; however, the samples do not accurately represent the various compartments of the gastrointestinal tract, especially the small intestine, where the nutrient absorption occurs. In addition, many different protocols are used for collecting the samples as well as for extracting bacterial DNA, making the cross-comparison of results difficult between studies. Thus, the complete sample process should be well standardized. Previous and most current studies have focused on the bacteria; however, bacteria only represent one part of the gut microbiota. Beyond bacteria, viruses, protists, archaea, and fungi are also present within the gut. Moreover, 16S sequencing measures the relative abundancies, but not the quantitative numbers that can be measured by different PCR or culture-based methods.

It would also be good to include subjective and objective endpoints related to mental and brain health in clinical trials, for example, measuring relevant biomarkers, brain imaging, or wearable devices to measure sleep. A thorough review of the preclinical datasets of the strain of interest as a guide for the best clinical model, populations, endpoints, and mechanisms needs to be thoroughly investigated. 

Depression and anxiety disorders are complex and linked to varied behavioral, cognitive, and physiological symptoms, possibly due to distinct molecular pathways. Furthermore, animal models of depression and anxiety can only reproduce certain features of these complex mood disorders. Of note, most of the randomized controlled clinical trials conducted in this field have investigated the impact of probiotics on (subclinical) depressive symptoms in healthy populations. Hence, most findings from these clinical trials cannot be extrapolated to patients with clinically diagnosed depression. The promise of probiotic treatments for MDD is complicated by the heterogeneous nature of both the gut microbiota composition and depressive symptoms in the clinical setting, depression subtypes, and probiotic formulations. However, despite these obstacles, probiotics have been shown to improve symptom severity in mood disorders and so there is promise that early interventions with probiotics to restore the gut microbiota composition could reduce the risk of the development of mood disorders such as depression and anxiety in later life. Further clinical investigations into the role of probiotics on mental and brain health and to investigate optimal probiotic composition, dosage, and duration of supplementation, employing high quality randomized, double-blind, placebo controlled clinical trials in different populations, are essential and certainly warranted. 

## Figures and Tables

**Table 1:** Common neurotransmitters and short chain fatty acids, their production, and functionality.

Neurotransmitter	Endogenous Production	Exogenous Production	Function	Remarks	References
Nitric oxide (NO)	Enteric inhibitory neurons	Enterobacteria, some lactobacilli and bifidobacteria, and some oral anaerobes	Gut motility, brain development, memory, and anti-anxiety	Enteric-produced NO does not play a role in anxiety	[38,39,40]
γ-aminobutyric acid (GABA)	GABA-ergic neurons	Some lactic acid bacteria and bifidobacteria	Neuroprotection, anti-diabetic, antioxidant, anti-inflammatory, anti-allergic, hepatoprotection, renoprotection, anti-depression, and anti-insomnia	Does not cross blood–brain barrier	[41,42,43]
Norepinephrine	Enteric nerve cells	*E. coli*, *Bacillus, Saccharomyces* spp., *S. marcescens* and *P. vulgaris*	Anti-inflammatory, anti-stress, and anti-anxiety	Does not cross blood–brain barrier	[44,45,46,47,48,49]
Dopamine	Central nervous system, various other tissues	*Bacillus* spp.	Locomotion, learning, working memory, cognition, and emotion	Does not cross blood–brain barrier	[46,49,50]
Acetylcholine	Cholinergic neurons	*L. plantarum*	Cognitive function and intestinal motility	Does not cross blood–brain barrier	[51,52,53]
Serotonin (5-hydroxytryptamine; 5-HT)	Serotonergic neurons mainly in the gut	*Candida, E. coli, Lc. lactis, L. plantarum, S. thermophilus, M. morganii, K. pneumoniae, H. alvei* and *Enterococcus* spp.	Regulation of mood, appetite, sleep, and cognitive function	Does not cross blood–brain barrier	[49,54,55,56,57]
Melatonin	Enterochromaffin cells in the gut	-	Regulation of circadian rhythm	Intestinal microbiota may be involved in breakdown	[57,58,59]
Indole	-	Actinobacteria, Firmicutes, Bacteroidetes, Proteobacteria, Fusobacteria, *Clostridium, Burkholderia, Streptomyces, Pseudomonas* and *Bacillus*	May influence emotional behavior	Crosses blood–brain barrier	[57,60]
Kynurenine and/kynurenic acid	Central nervous system, various other tissues	*B. infantis*	Associated with depression and schizophrenia	Increased kynurenic acid: kynurenine is neuroprotective. Both can cross blood–brain barrier	[57,61,62]
Quinolinic acid	Epithelial cells and intestinal immune cells	-	Associated with depression	Neurotoxic. Does not cross blood–brain barrier. May be blocked by *L. helveticus* and *B. longum*	[57,61,63,64]
Histamine	Mast cells and other immune cells	Certain lactic acid bacteria fermented foods	Mediates arousal, attention, and reactivity	Does not cross blood–brain barrier	[56,65,66,67]
Short chain fatty acids (SCFA) *	Muscle tissue	Most anaerobes in the gut	Regulate inflammation, appetite, depression, and gut motility	Crosses blood–brain barrier	[68,69,70,71,72,73,74,75,76,77,78,79,80,81]

* Short chain fatty acids are not neurotransmitters. However, as they may modulate the levels of neurotransmitters, they are included here.

## Data Availability

No new data was generated in writing this review.
